# PARENTHOOD AND MARRIAGE WITHIN SAME-SEX COUPLES: AN ANALYSIS USING SURVEY, TIMELINE, AND IN-DEPTH INTERVIEW DATA

**DOI:** 10.1093/geroni/igz038.1534

**Published:** 2019-11-08

**Authors:** Mieke Thomeer, Allen J LeBlanc, David M Frost

**Affiliations:** 1 University of Alabama-Birmingham, Birmingham, Alabama, United States; 2 San Francisco State University, San Francisco, California, United States; 3 University College London, London, England, United Kingdom

## Abstract

In this study, the authors analyze data from Project SHARe, a mixed-methods study of same-sex couples in two cities, Atlanta and San Francisco. This study considers both past experiences of parenthood and marriage among same-sex couples, as well as what same-sex couples anticipate will occur regarding their own future experiences of parenthood and marriage, based on analysis of survey responses, timeline data, and in-depth interviews.

